# HEALTH IMPLICATIONS OF SOCIAL ROLES AND ROLE TRANSITIONS IN MIDLIFE AND LATER LIFE

**DOI:** 10.1093/geroni/igz038.2933

**Published:** 2019-11-08

**Authors:** Kelly E Cichy, Athena Koumoutzis

**Affiliations:** Kent State University, Kent, Ohio, United States

## Abstract

Demographic and social trends shape the timing, nature, and implications of social roles and transitions. With increased life expectancy and a changing world, expectations for work and retirement and the need for informal and formal caregiving continue to evolve. Families are also more heterogeneous and the population is becoming increasingly more racially/ethnically diverse. These changes underscore the need for research that focuses on the varied social roles individuals occupy in midlife and later adulthood and the implications of these roles for health and well-being. The current symposium features research that explores multiple roles, including romantic partner, grandparent, and employee/retiree, caregiver/care recipient while attending to individual differences in how these roles and transitions are associated with physical and mental health outcomes. Garcia, Donnelly, and Umberson utilize dyadic diary data from midlife men and women in gay, lesbian, and heterosexual marriages to consider how exposure and reactivity to daily stress varies across union types. Rickenbach and colleagues examine longitudinal changes in health and well-being associated with being a caregiving and non-caregiving grandparent. Cichy and Koumoutzis examine racial differences in the associations between providing care to a spouse/parent and daily health and well-being among African Americans and European Americans. Savla, Roberto, and Sands classify community-living older adults based on their care needs while considering the type of care they receive, predictors of this care, and its implications for care recipients’ health. Finally, Stawski and colleagues examine how mental, physical, and cognitive health change as a function of the transition to and through retirement.

